# The influence of ultrasound and adenosine 5′-monophosphate marination on tenderness and structure of myofibrillar proteins of beef

**DOI:** 10.5713/ajas.18.0780

**Published:** 2019-03-07

**Authors:** Ye Zou, Heng Yang, Muhan Zhang, Xinxiao Zhang, Weimin Xu, Daoying Wang

**Affiliations:** 1 Institute of Agro-product Processing, Jiangsu Academy of Agricultural Sciences, Nanjing 210014, China; 2 College of Food Science and Engineering, Nanjing University of Finance and Economics, Nanjing 210046, China

**Keywords:** Ultrasound, Adenosine 5′-Monophosphate, Tenderization, Myofibrillar Fragmentation Index, Synchronous Fluorescence Spectra

## Abstract

**Objective:**

The aim was to investigate the influence of ultrasound and adenosine 5′-monophosphate (AMP) marination (UAMP) on tenderness and structure of myofibrillar proteins of beef.

**Methods:**

Five groups, the untreated meat (Control), deionized water marination (DW), ultrasound followed by DW (UDW), AMP marination (AMP), and ultrasound followed by AMP (UAMP) were studied. Myofibrillar fragmentation, cooking loss, shear force, thermograms, histological observation of meats and myofibrillar proteins properties were investigated in these different treatments.

**Results:**

The results showed that UAMP significantly increased myofibrillar fragmentation index from 152 (Control), 231 (AMP), and 307 (UDW) to 355 (p<0.05), respectively. The lowest cooking loss, shear force and peak denaturation temperature were observed in UAMP. In histological observation, UDW and UAMP had more fragmented muscular bundles than the others. Furthermore, a drastic increase in α-helix and decrease in β-sheet of myofibrillar proteins was observed in UAMP, implying the disaggregation of protein samples. The synchronous fluorescence spectra of myofibrillar proteins in UAMP suggested the combination of ultrasound and AMP could accelerate the unfolding molecular structure and destroying hydrophobic interactions. The results of circular dichroism and synchronous fluorescence spectra for myofibrillar proteins coincided with the microstructures of beef.

**Conclusion:**

The results indicate that ultrasound combined with AMP improved meat tenderness not only by disruption in muscle integrity, increasing water retention, but also altering their spatial structure of myofibrillar proteins.

## INTRODUCTION

The effects of tenderization procedures on other qualities such as color and flavor are different depending on methods [1]. Among these meat sensory properties, tenderness is one of the principal contributory elements for customer acceptability. Meat tenderness is influenced by several factors, including biological and extrinsic factors (calcium salt, phosphate salt, organic acid, etc.). In practical application, ageing is a critical procedure to tenderize meat [2]. Therefore, a variety of mechanical, physical, biological and chemical approaches have been used by meat processors to attain tender meat [3–5]. Different from conventional ageing, the most common usage of tumbling and injection has lowered product costs and raised the volume of production. However, injection might destroy the appearance of meat products and rapid tumbling can result in heat production and poor quality products. Alternative methods for meat tenderization are still needed.

Low frequency ultrasound (20 to 100 kHz) is an emerging technology that can induce chemical, biological and mechanical actions in meat [6]. The micro-channels, micro-stirring, high-temperature, high-pressure and free radicals caused by ultrasound are conducive to accelerating material transport and causing textural or structural transformations in meat and meat products [7]. Studies have indicated that ultrasonication gives rise to increased thickness of filaments [8]. Ozuna et al [9] deem that ultrasound treatment can cause changes in the surface as well as the interior microstructural of meat during the curing process. Ultrasound combined with other processes are considered to be effective in improving overall meat quality, for example margination could alter the physic-chemical and functional properties in materials [10]. Xiong et al [11] found that contribution of mechanical damage by the effects of ultrasound treatment and the release of endogenous proteases tenderized meat and increased its water holding capacity. Additionally, combined processing, for instance ultrasound in combination with exogenous enzymes and different chemicals, could have a a synergistic effect on meat tenderness [12]. Nevertheless, the shape, colour and luster, and structures of meat products changed dramatically after enzyme treatment.

Adenosine 5′-monophosphate (AMP) is an endogenous purine nucleotide, which is present in all animals and is involved in energy metabolism [13]. In the process of foodstuff processing, AMP is frequently added to poultry, fish, meat, and vegetable soups as a flavoring agent, or as a food ingredient for specific nutrition [14]. Additionally, a significant increase of actin can be detected following AMP treatment of protein solutions extracted from animal bones. Furthermore, AMP can promote the dissociation of actomyosin, which can promote meat tenderness. However, few studies have evaluated the combined impact of ultrasound and AMP marinating (UAMP) on meat tenderness. Therefore, the aim of this study was to evaluate the effects of UAMP on beef tenderness. The effect of the combined treatment on myofibrillar fragmentation, cooking loss, shear force, thermograms, histological observation of meats and myofibrillar proteins (MPs) properties were studied and compared with those meats of ultrasound or AMP alone and control to further elucidate the mechanism of tenderization for the combined treatment.

## MATERIALS AND METHODS

### Material

*Semimembranosus* muscles (Angus×Hereford, 51 steers, 18 months of age, ~2.5 kg/steer) were obtained from a local meat market. AMP and tris (hydroxymethyl) metyl aminomethane were purchased from Sigma Chemical Co. (St. Louis, MO, USA). All the chemicals and reagents in this study were of analytical grade.

### Sample preparation

The beef meats were vacuum packed and aged at 2°C±2°C for 6 days post mortem. The muscle was removed of visible fat, fibrillar and areolar connective tissue and cut into cubes with a mean mass of 50±2 g. The raw meat was randomly divided into five groups and stored at −18°C. Before treatment, the raw beef was thawed at 4°C for about 12 h. In this experiment, raw meat that had no treatment during 30 min of conditioning was used as Control (n = 6). The second group (DW, n = 6) was marinated with deionized water (pH 7.0, 250 mL) for 30 min. The third group (UDW, n = 6) was treated with ultrasound for 5 min (20 kHz, 200 W, 15.6 W/cm^2^, ultrasound solution is deionized water) and then marinated in deionized water (pH 7.0, 250 mL) for 25 min. The fourth group (AMP, n = 6) was marinated with 32 mM AMP solution (pH 8.0, 250 mL) for 30 min, and the last group (UAMP, n = 6) was treated with ultrasound for 5 min (20 kHz, 200 W, 15.6 W/cm^2^, ultrasound solution is deionized water) followed by AMP (32 mM, pH 8.0, 250 mL, 25 min). All samples were treated at 20°C. Duplicate measurements per sample were taken for all analysis. After these treatments, the samples were frozen by liquid nitrogen and then kept in a −40°C refrigerator for further use.

### Myofibrillar fragmentation index

Myofibrillar fragmentation index (MFI) was determined based on the method of Wang et al [15] with slight modifications. The above uncooked beef (2.0 g) was homogenized in phosphate buffer (0.1 M KCl, 1 mM NaN_3_, 7 mM KH_2_PO_4_, 18 mM K_2_HPO_4_, 1 mM MgCl_2_, 1 mM ethylene diamine tetraacetic acid, pH 7.0, 20 mL, 4°C) in a falcon tube. The homogenate was filtered using mesh strainers to remove connective tissue and centrifuged at 4°C. The pellets of myofibrils were collected and centrifuged again with added phosphate buffer. The sediment was re-suspended in buffer (10 mL) and centrifuged again. This process was repeated, and the pellet was finally re-suspended in cold buffer solution. The concentration of protein was diluted to 0.5 mg/mL with the bicinchoninic acid (BCA kit, A045-3, Jiancheng Institute of Biotechnology, Nanjing, China) and measured at 540 nm in a UV spectrophotometry for 5 times. The mean reading of absorbance was multiplied with 200 as MFI.

### Cooking loss

Beef samples were cooked in plastic bags (48±2 g, width× length, 10 cm×15 cm) individually in a water bath kettle set at 95°C until the internal meat temperature reached 75°C. After cooking, the samples were cooled immediately in ice-water bath to the internal temperature of room temperature (25°C) and wiped with filter paper to remove excess water on the surface and weighted. Cooking loss was calculated as reported by Zou et al [1].

### Shear force

Meullenet-Owens razor shear force (MORS) method uses an extremely sharp blade with a certain size and connects it to physical property analyzer (texture analyzer) for cutting/shearing test. MORS text of the cooked samples was measured through a texture analyzer (TVT-300XP, TexVol Instruments, Viken, Sweden). A perpendicular strip (10×10×50 mm) of 1 cm^2^ area was cut from each muscle sample. The test speed was set at 150 mm/min and the trigger force was 200 g, and 6 technical replicates per sample were tested [7].

### Differential scanning calorimeter of meat

Denaturation of proteins in the untreated and ultrasonic treated meat (uncooked sample) was analyzed by differential scanning calorimeter (DSC). DSC data were obtained by a NETZSCH DSC (STA-449C, NETZSCH Scientific Instruments Trading Ltd., Chemnitz, Germany). Analysis was done immediately after treatments, unless otherwise stated. A blank was a sealed empty pan. Uncooked beef samples (6.0 mg) were heated at a rate of 10°C/min from 30°C to 150°C, respectively. The integrated area above the endothermic peak from the thermal curve stands for the enthalpy (J/g). At least three runs average of the calorimetric results for samples were determined, respectively.

### Histological analysis

Cross and longitudinal sections were cut from each sample. Tissues (uncooked samples) were fixed with phosphate-buffered (10%, pH 6.9 to 7.1) formaldehyde for 1 day. Blocks of tissue (20 mm×10 mm×5 mm) were chipped from the surface of the treated meat samples. After the dehydration, paraffin embedding, sectioning, and dyeing treatment, the microstructure of the beef myofibrils were observed with an optical microscope (Eclipse E100, Nikon, Kyoto, Japan). Pictures were recorded with digital video camera (DS-U3, Nikon, Japan).

### Analyses of myofibrillar proteins

#### Sodium dodecyl sulfate-polyacrylamide gel electrophoresis

MPs were extracted according to the methods of Wang et al [16]. Beef (2 g, uncooked samples) was added to 8 times of separation buffer (0.1 M KCl, 2 mM MgCl_2_, 1 mM ethylene diamine tetraacetic acid, 0.5 mM dithiothreitol, 10 mM K_2_HPO_4_, pH 7.0) and homogenized in ice bath (10,000×*g*, 10 s, 3 times). After homogenizing, the samples were centrifuged (2,000×*g*, 20 min). Then the supernatant was discarded, and the pellet was collected. And then the above steps were repeated three times and the purified MP precipitation was obtained. The precipitates were dissolved in appropriate buffer (0.6 M KCl, 10 mM K_2_HPO_4_, pH 6). The concentration of protein was diluted to 0.5 mg/mL with bicinchoninic acid (BCA kit, A045-3, Jiancheng Institute of Biotechnology, China). The MPs were analyzed by sodium dodecyl sulfate-polyacrylamide gel electrophoresis (SDS-PAGE) following the description by Wang et al [16] with some modifications. A 10% resolving gel and a 4% stacking gel were prepared. Briefly, 0.2% MPs solution after heating (100°C, 5 min) were separated in an electrophoresis device (PowerPac, Bio-Rad, Hercules, Singapore). Gels were stained with coomassie brilliant blue-R250 and destained with ethyl alcohol:acetic acid:DW (50:10:40, v/v/v) acetic acid in DW.

#### Circular dichroism spectral analysis

The spectra of MPs from beef were recorded in the range from 190 to 250 nm using a circular dichroism (CD) spectropolarimeter (Jasco 1500, Jasco Corp., Tokyo, Japan) at 25°C. An optical path of quartz CD cuvette was 1.0 mm [17]. The sample concentrations of MPs were 200 μg/mL for chemical analysis, and the corresponding phosphate buffer solvent (20 mM, pH 7.0) without MPs was used as blank to provide a spectral background. The scan rate, response time and band width were set at 100 nm/min, 0.50 s and 2.0 nm, respectively. Three scanning acquisitions were accumulated and averaged to yield the last circular dichrogram.

#### Synchronous fluorescence spectra

In the fluorescence spectrophotometer of MPs, the wavelength difference Δλ between emission wavelength λ_em_ and the excitation wavelength λ_ex_ was changed (λ_em_ = λ_ex_+Δλ). The fluorescence spectra of tyrosine (Δλ = 15 nm) and tryptophan (Δλ =60 nm) chromophore were obtained from MPs with different treatments when the emission wavelength was from 200 to 500 nm.

### Statistical analysis

All determinations were carried out with at least triplicate samples. The results in this study are presented as the mean± standard deviation. Analysis of variance with p<0.05 by means of SPSS 19.0 was employed to assess the effect of different treatment with significant difference. The Duncan’s multiple range tests were used to locate differences. All analyses including figures and calculations were conducted with the Origin Pro 9.0.

## RESULTS AND DISCUSSION

### Myofibrillar fragmentation index

**Figure 1:**
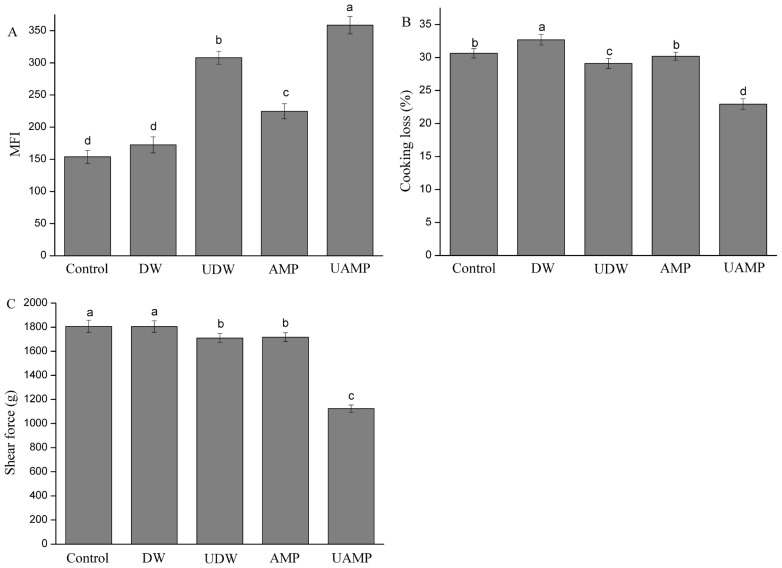
(A) The change of myofibrillar fragmentation index values (MFI) in five groups during conditioning. (B) Cooking loss of beef meat samples treated with different treatment. (C) The changes of shear force in five groups. Different letters indicated that there were significant differences in different treatments (p<0.05). DW, deionized water marination; UDW, ultrasound followed by deionized water; AMP, adenosine 5′-monophosphate treatment; UAMP, ultrasound followed by adenosine 5′-monophosphate marination.

The MFI is a helpful indicator to measure the degradation degree of myofibril and tenderness in meat. The increased MFI value may be correlated with the breaking down of the MPs into segments or loss of Z-disks which occurred during treatment. The MFI values of five groups are shown in Figure 1A. Compared to Control, the value of MFI in other groups significantly increased (p<0.05) except for that of DW. Furthermore, UAMP had the highest MFI values followed by UDW and AMP. The result indicated that UAMP could enhance the MFI value over ultrasound or AMP treatment alone. The MFI values of AMP were higher than those of Control and DW. Wang et al [18] reported that AMP marination exhibited a significant increased MFI over the untreated duck breast, which was consistent with our results. The reason UAMP has a remarkable ability to increase MFI might be due to the ultrasound causing mechanical and acoustic cavitation, and turbulence. This may have led to the exposure of the hydrophobic cores, which are buried inside the molecules of the protein and disrupted the interactions between the local sequences of amino acids and between the different parts of the protein molecule. The combined effect could cause biological transformations of the beef tissue, and accelerated AMP permeation and chemical reactions inside the meat, which agrees with the reports of Kang et al [19].

### Cooking loss

The moisture data of Control, DW, UDW, AMP, and UAMP beef was 72.25%, 73.06%, 72.72%, 72.54%, and 73.13%, respectively. There was no significant difference between these different treatments. The cooking loss of the meat samples with different treatments is shown in Figure 1B. The DW had the highest cooking loss among the treatments (p<0.05). There were no significant differences in cooking loss between AMP and Control, which was different from the study of Wang et al [20]. This phenomenon might be due to the short treatment time (30 min), while the curing time of AMP was 10 h in the study of Wang et al [20]. The cooking loss of UDW was significantly lower than that of Control and DW. Ultrasound treatment could not only destroy the structure of MP and release a large amount of salt-soluble protein, but also promote the enrichment of salt-soluble protein at the surface of muscle and enhance the ability of muscle surface to prevent water spillover. The cooking loss significantly decreased in UAMP over that in UDW (p<0.05). This indicated that ultrasound treatment could improve the cooking yield in AMP solution more than in DW. With the penetration of 32 mM AMP, phosphate or other charged particles bound to beef myofibrils could induce static shielding and increase the electrostatic repulsion between filaments, which caused myofibrils expanding and increase water retention [21].

### Shear force

Tenderness is one of the most indispensable meat qualitative attributes for the consumer of meat and meat products. The changes of shear force in five groups are shown in Figure 1C. Among five treatments, the shear force of DW and Control did not have significant differences (p>0.05). Shear force of UDW and AMP were significantly lower than that of those two treatments (p<0.05). Wang et al [20] found that duck breast meat became tender with AMP marination (40 mM, 10 h, 5°C), which induced the breakdown of cellular architecture and disruption of the integrity of myofibrillar structure. These findings are coincidental with the research findings of Siró et al [22] who observed lower shear force values than the control when meat was treated with the appropriate ultrasonic intensity. However, McDonnell et al [23] found that there was no change in hardness of beef when samples were treated by ultrasound-assisted curing, but Ozuna et al [9] demonstrated that the hardness of pork meat increased when ultrasound treatment was applied. This result of UDW could be attributed to the formation of localized hot spots upon collapse of bubbles, and to the generation of microjets, shear forces, shock waves and turbulence which were induced by the acoustic cavitation. The cavitation not only increased the water content in beef but also produced the physical disruption and degradation of meat protein by ultrasound treatment [6]. Additionally, the lowest shear force values for UAMP were observed in this study. These results seem to confirm the positive effect of ultrasound assisting the penetration of AMP solution inside meat pieces because of the increase of spaces between myofibrils.

### Histology

**Figure 2:**
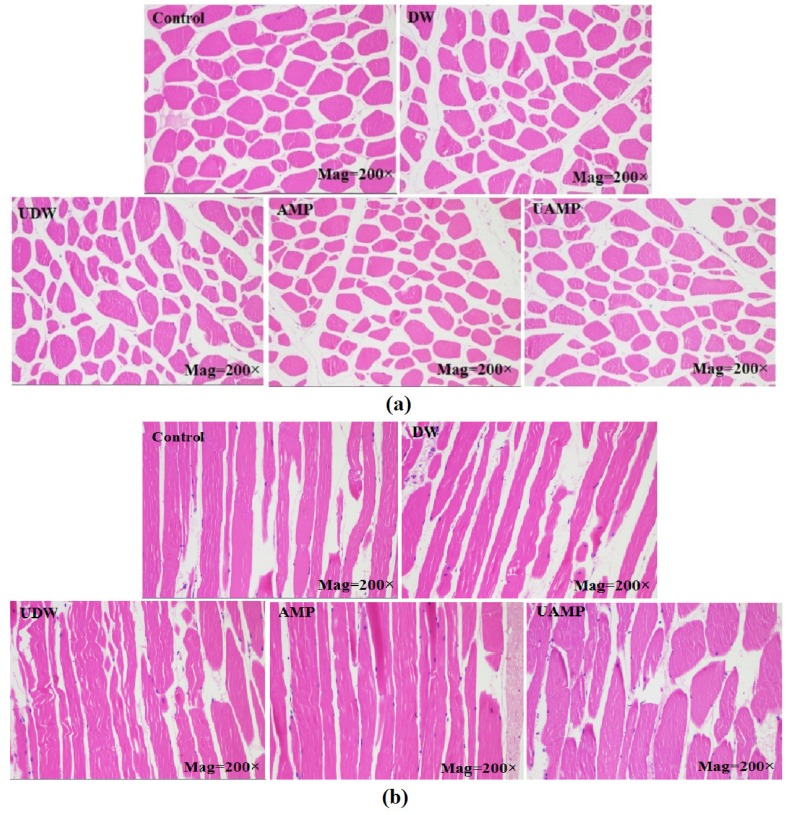
Light microscopy photographs of transverse and longitudinal sections of beef meat in different treatments. (a) Cross sectional microstructure; (b) vertical sectional microstructure. DW, deionized water marination; UDW, ultrasound followed by deionized water; AMP, adenosine 5′-monophosphate treatment; UAMP, ultrasound followed by adenosine 5′-monophosphate marination.

The cross (a) and longitudinal (b) sectional microstructures of meats in different groups are presented in Figure 2. The samples exhibited an intact cell membrane with uniform shape and narrow intervals in Control and DW, while AMP showed a small cross-sectional area compared to that of control. The reason might be the short marination time (30 min) for AMP group. The myofibrillar spacing was smaller in AMP, UAMP, and UDW than Control. However, samples in UDW and UAMP had wider fragmented cross section. On the other hand, the loss of structural integrity, tissue damage and myofibrillar swelling was seen in a longitudinal sections of UDW and UAMP as compared to other treatment. Dolatowski [24] found that ultrasound pretreatment for horseflesh combined with curing led to destruction myofibrils and rupture of cellular structures. Siró et al [22] manifested that ultrasound with 20 kHz and 2 to 4 W/cm could breakdown the Z-line and increase the wide channels between muscle myofibrils, indicating that ultrasonication for 30 to 180 min may result in an irregular nebulin network. In this study, application of UAMP resulted in damage to histiocytes, which separated beef fibers with larger intracellular spaces. This result might be owing to more water in the muscle fibers interspaces in UAMP than single ultrasound or AMP treatments. Extensive changes in the microstructure of muscle fibers have induced improvement of beef tenderness and acceptability.

### Differential scanning calorimeter of meat

The peak temperature (T_d_) usually reflects the stability of biological macromolecule structure, while enthalpy (ΔH) is the required energy to arouse a conformational transformation in DSC. The Control sample showed two endothermic peaks at 66.07°C and 79.30°C (Table 1), identified as myosin and actin, respectively in the samples. Shifts in the T_d_ of the proteins from their control to lower T_d_ and lower energy required denaturing signified by destabilisation of protein structure in UDW and UAMP groups. This might be due to the unfolding of protein molecule, due to the disruption of intra-molecular bonds of myosin. Furthermore, the denaturation temperature peak of myosin and actin reduced to 63.30°C and 76.25°C in UAMP, respectively. Owing to low ion concentrations, beef filament swelled in other studies [25]. This expansion of beef filaments might weaken the bond between the F-actin polymer and myosin molecule, thus lowering denaturation temperature of proteins in AMP treatments. The lowest T_d_ and ΔH were observed in the UAMP treatment. The result demonstrated that ultrasound treatment could unfold the protein molecule, disrupt intramolecular bonds (such as hydrophobic interactions) and cause some degree of protein degeneration. The decline in T_d_ and ΔH was due to molecular transformation in protein structure in the different treatments.

### Effect of different treatment on the molecular structure of myofibrillar protein

#### Sodium dodecyl sulfate-polyacrylamide gel electrophoresis

**Figure 3:**
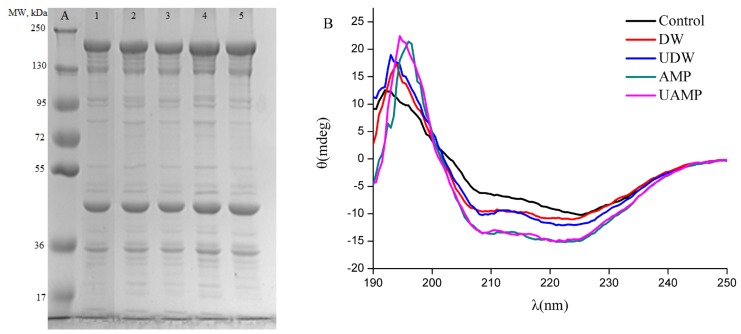
Effect of different treatment on the molecular structure of myofibrillar protein. (A) Sodium dodecyl sulfate-polyacrylamide gel electrophoresis (SDS-PAGE) profiles of myofibrillar proteins from five groups. Line A, standard protein marker; Line 1, Control; Line 2, DW; Line 3, UDW; Line 4, AMP; and Line 5, UAMP. (B) Circular dichroism (CD) Spectra of myofibrillar proteins from five groups. DW, deionized water marination; UDW, ultrasound followed by deionized water; AMP, adenosine 5′-monophosphate treatment; UAMP, ultrasound followed by adenosine 5′-monophosphate marination.

Figure 3A shows the SDS-PAGE results of MPs obtained from different treatments. Different polypeptides with known molecular weights (MW) in the MPs profiles were myosin heavy chain (MHC, ~200 kDa), actin (~44 kDa) and troponin-T (~35 kDa). The results in SDS-PAGE profiles indicated that the distribution of MW of protein isolates had no significant change due to treatment by ultrasound or AMP or their combined action as compared to Control. Similar observations were reported by O’Sullivan [26] on animal (fish gelatin, bovine gelatin and egg white protein, 20 kHz and 2 min) for ultrasound treatment. In contrast, MW of protein isolates was reduced by ultrasound treatment of the sun flower protein isolates [27]. The difference in these results might be due to various processing conditions and also different sources of protein. Figure 3A shows a high intensity of MHC, actin and troponin-T induced by AMP and UAMP compared to that of other treatments (p<0.05), which might be due to the dissociation of actomyosin and tropomyosin by higher ionic strength [28]. In particular, the increase in intensities of actin and troponin-T might be due to the synergistic effect of higher turbulence, mechanical shear and cavitation from ultrasound with higher ionic strength on the MPs, which resulted in cleaved molecular structure of polymers proteins.

#### CD spectra

Our results indicated that the CD spectra of MPs from DW are different from the Control (Figure 3B), suggesting changes in secondary structure in these two groups. When UDW or AMP was given, the intensity of the CD spectra was appreciably strengthened, suggesting a visible transformation from β-sheet to α-helices by UDW or AMP alone. By calculations from the program CONTINLL (a software for calculating protein secondary structure), UDW and AMP caused 26.8% and 73.7% increase in α-helical fraction, and 16.6% and 74.3% decline in β-sheet content compared with the control as seen in Table 2, indicating marked change in secondary structure of MPs. The results indicated that α-helical content of MPs, especially myosin rod, could gradually undergo folding under the effect of either ultrasound or AMP, which was similar with the report of Saleem and Ahmad [29]. The increase and decrease in α-helix and β-turn content was observed in UAMP in the same treated time as the present study, respectively, implying that ultrasound and AMP have synergistic effects. The results of this study was in agreement with the research of Huang et al [30] who reported the increase of α-helical and the loss of β-sheet accounted for disaggregation of soybean protein. The result of UAMP might be due to AMP marination and mechanical/cavitation destruction of ultrasound.

#### Synchronous fluorescence spectra

**Figure 4:**
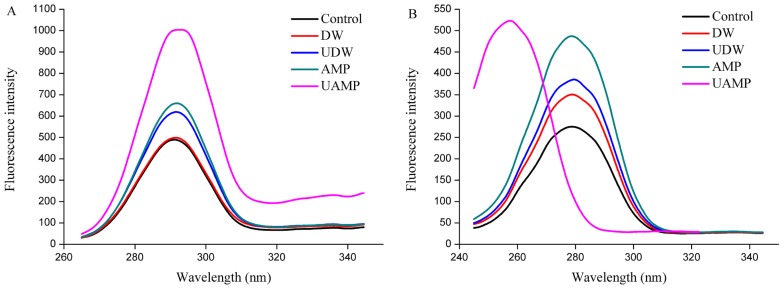
Synchronous fluorescence spectra of myofibrillar proteins from five groups. (A) synchronous fluorescence spectra of Tyr; (B) synchronous fluorescence spectra of Trp.

Trp and Tyr are the main fluorescein groups in proteins. When the polar environment of aromatic amino acids increased, the fluorescence peak is red shifted, indicating that the protein molecules were stretched. Whereas, when the non-polar environment moves, the fluorescence peak is blue shifted. Fluorescence intensity of MPs increased in UDW, AMP, and UAMP (Figure 4A), while the peak shape is unchanged in synchronous fluorescence spectra of Tyr, indicating that single AMP and UDW and its combination did not destroy the protein primary structure. Besides, the fluorescence intensity of MPs in UAMP was the highest followed by that in AMP and UDW. Additionally, the largest increases of fluorescence intensity in UAMP suggested its structural changes were the most remarkable compared to that in single UDW and AMP treatments. The result also indicated that the combination of ultrasound and AMP could accelerate the unfolding of the molecular structure, destroying hydrophobic interactions, and thus increasing the fluorescence intensity of MPs. The cavitation effect, mechanical effect and super mixing effect caused by UAMP may extend the protein molecules and expose different chromophores, thus changing the intensity of fluorescence of MPs. It was interesting in synchronous fluorescence spectra of Trp that, after ultrasound treatment, the maximum emission peak of fluorescence spectrum from UAMP was blue shifted. This indicated that UAMP could expose hydrophobic groups in MPs in Figure 4B. The result is consistent with the result of Figure 4A. Thus, the changes of the spatial structure of MPs provide the basis for understanding the mechanism for tenderization beef by UAMP.

## CONCLUSION

UAMP improved meat tenderness not only by disruption in muscle integrity and increasing water retention, but by also altering their spatial structure of MPs, suggesting that AMP marination and ultrasound treatment have synergistic effects in tenderization. The alternations of CD and synchronous fluorescence spectra of MPs suggests that they were dissociated. Therefore, the combination of ultrasound and AMP appears to be a promising and efficient tenderizing technique in the meat industry.

## Figures and Tables

**Table 1:** Thermal denaturation temperature and denaturation enthalpy of beef in different treatments

Treatments	T_1_ (°C)	ΔH_1_ (J/g)	T_2_ (°C)	ΔH_2_ (J/g)
Control	66.07±0.4^a^	1.553±0.02^a^	79.30±0.5^a^	0.434±0.04^b^
DW	65.89±0.5^a^	1.572±0.01^a^	79.22±0.4^a^	0.478±0.03^a^
UDW	65.19±0.3a,b	1.317±0.02^b^	78.01±0.5a,b	0.471±0.02^a^
AMP	64.12±0.4^b^	1.315±0.03^b^	77.82±0.6^b^	0.366±0.04^c^
UAMP	63.30±0.3^c^	1.279±0.02^b^	76.25±0.4^c^	0.315±0.01^d^

Data are expressed as mean±standard deviation (n = 3).

DW, deionized water marination; UDW, ultrasound followed by deionized water; AMP, adenosine 5′-monophosphate treatment; UAMP, ultrasound followed by adenosine 5′-monophosphate marination.

a–d Means in the same column with different letters mean significantly different (p<0.05).

**Table 2:** Secondary structure contents of myofibrillar protein with different treatments

Method	α-Selix (%)	β-Sheet (%)	β-Turn (%)	Random coil (%)
Control	18.3±0.16^e^	45.2±0.35^a^	12.3±0.09^e^	24.2±0.23^c^
DW	20.5±0.21^d^	42.4±0.31^b^	13.3±0.12^d^	23.9±0.18^d^
UDW	23.2±0.17^c^	37.7±0.28^c^	14.3±0.14^c^	24.8±0.32^c^
AMP	31.0±0.19^b^	15.4±0.08^d^	27.4±0.27^b^	26.2±0.11^b^
UAMP	31.8±0.23^a^	11.6±0.15^e^	29.3±0.22^a^	27.3±0.26^a^

Data are expressed as mean±standard deviation (n = 3).

DW, deionized water marination; UDW, ultrasound followed by deionized water; AMP, adenosine 5′-monophosphate treatment; UAMP, ultrasound followed by adenosine 5′-monophosphate marination.

a–e Means in the same column with different letters mean significantly different (p<0.05).

## References

[1] Zou Y, Zhang K, Bian H (2018). Rapid tenderizing of goose breast muscle based on actomyosin dissociation by low-frequency ultrasonication. Process Biochem.

[2] Kim YH, Ma D, Setyabrata D (2018). Understanding postmortem biochemical processes and post-harvest aging factors to develop novel smart-aging strategies. Meat Sci.

[3] Barekat S, Soltanizadeh N (2017). Improvement of meat tenderness by simultaneous application of high-intensity ultrasonic radiation and papain treatment. Innov Food Sci Emerg Technol.

[4] Yusop SM, O’Sullivan MG, Kerry JF, Kerry JP (2012). Influence of processing method and holding time on the physical and sensory qualities of cooked marinated chicken breast fillets. LWT - Food Sci Technol.

[5] Warner RD, McDonnell CK, Bekhit AED (2017). Systematic review of emerging and innovative technologies for meat tenderisation. Meat Sci.

[6] Alarcon-Rojo AD, Janacua H, Rodriguez JC, Paniwnyk L, Mason TJ (2015). Power ultrasound in meat processing. Meat Sci.

[7] Zou Y, Yang H, Li P (2019). Effect of different time of ultrasound treatment on physicochemical, thermal, and antioxidant properties of chicken plasma protein. Poult Sci.

[8] Got F, Culioli J, Berge P (1999). Effects of high-intensity high-frequency ultrasound on ageing rate, ultrastructure and some physico-chemical properties of beef. Meat Sci.

[9] Ozuna C, Puig A, García-Pérez JV, Mulet A, Cárcel JA (2013). Influence of high intensity ultrasound application on mass transport, microstructure and textural properties of pork meat (*Longissimus dorsi*) brined at different NaCl concentrations. J Food Eng.

[10] Turantaş F, Kılıç GB, Kılıç B (2015). Ultrasound in the meat industry: General applications and decontamination efficiency. Int J Food Microbiol.

[11] Xiong GY, Zhang LL, Zhang W, Wu J (2012). Influence of ultrasound and proteolytic enzyme inhibitors on muscle degradation, tenderness, and cooking loss of hens during aging. Czech J Food Sci.

[12] Barekat S, Soltanizadeh N (2017). Improvement of meat tenderness by simultaneous application of high-intensity ultrasonic radiation and papain treatment. Innov Food Sci Emerg Technol.

[13] Pascal JM (2008). DNA and RNA ligases: structural variations and shared mechanisms. Curr Opin Struct Biol.

[14] Khetra Y, Kanawjia SK, Puri R (2016). Selection and optimization of salt replacer, flavour enhancer and bitter blocker for manufacturing low sodium Cheddar cheese using response surface methodology. LWT - Food Sci Technol.

[15] Wang D, Zhang M, Deng S (2016). Postmortem changes in actomyosin dissociation, myofibril fragmentation and endogenous enzyme activities of grass carp (*Ctenopharyngodon idellus*) muscle. Food Chem.

[16] Wang J, Yang Y, Tang X, Ni W, Zhou L (2017). Effects of pulsed ultrasound on rheological and structural properties of chicken myofibrillar protein. Ultrason Sonochem.

[17] Zou Y, Bian H, Li P (2018). Optimization and physicochemical properties of nutritional protein isolate from pork liver with ultrasound-assisted alkaline extraction. Anim Sci J.

[18] Wang DY, Deng SY, Zhang MH (2015). Optimization of the tenderization of duck breast meat by adenosine 5′-monophosphate (AMP) using response surface methodology. J Poult Sci.

[19] Kang D, Wang A, Zhou G, Zhang W, Xu S, Guo G (2016). Power ultrasonic on mass transport of beef: Effects of ultrasound intensity and NaCl concentration. Innov Food Sci Emerg Technol.

[20] Wang DY, Deng SY, Zhang MH (2016). The effect of adenosine 5′-monophosphate (AMP) on tenderness, microstructure and chemical-physical index of duck breast meat. J Sci Food Agric.

[21] Gudjónsdóttir M, Arason S, Rustad T (2011). The effects of pre-salting methods on water distribution and protein denaturation of dry salted and rehydrated cod – A low-field NMR study. J Food Eng.

[22] Siró I, Vén C, Balla C, Jónás G, Zeke I, Friedrich L (2009). Application of an ultrasonic assisted curing technique for improving the diffusion of sodium chloride in porcine meat. J Food Eng.

[23] McDonnell CK, Lyng JG, Allen P (2014). The use of power ultrasound for accelerating the curing of pork. Meat Sci.

[24] Dolatowski ZJ (1988). Ultrasonics. 2. Influence of ultrasonics on the microstructure of muscle-tissue during curing. Fleischwirtschaft.

[25] Graiver N, Pinotti A, Califano A, Zaritzky N (2006). Diffusion of sodium chloride in pork tissue. J Food Eng.

[26] O’Sullivan J, Murray B, Flynn C, Norton I (2016). The effect of ultrasound treatment on the structural, physical and emulsifying properties of animal and vegetable proteins. Food Hydrocolloid.

[27] Malik M, Sharma H, Saini C (2017). High intensity ultrasound treatment of protein isolate extracted from dephenolized sunflower meal: Effect on physicochemical and functional properties. Ultrason Sonochem.

[28] Thorarinsdottir KA, Arason S, Sigurgisladottir S, Valsdottir T, Tornberg E (2011). Effects of different pre-salting methods on protein aggregation during heavy salting of cod fillets. Food Chem.

[29] Saleem R, Ahmad R (2016). Effect of low frequency ultrasonication on biochemical and structural properties of chicken actomyosin. Food Chem.

[30] Huang L, Ding X, Dai C, Ma H (2017). Changes in the structure and dissociation of soybean protein isolate induced by ultrasound-assisted acid pretreatment. Food Chem.

